# A Rare Case of Spontaneous Bilateral Subcapsular Renal Hematoma

**DOI:** 10.1177/2324709620918098

**Published:** 2020-05-13

**Authors:** Tushar Bajaj, Ngon Trang, Faisal Nasrawi, Sabitha Eppanapally

**Affiliations:** 1 UCLA—Kern Medical, Bakersfield, CA, USA; 2 Ross University, Miramar, FL, USA

**Keywords:** critical care, renal, kidney, subcapsular, hematoma, education

## Abstract

Spontaneous bilateral renal subcapsular hematoma is a rare condition. On literature review, only 2 case reports have elucidated possible etiologies for such a presentation; however, no definite conclusions have been made. We present a rare case of a 52-year-old female with diabetes mellitus type 2, chronic kidney disease stage 4, hypertension, hyperlipidemia, prior traumatic brain injury via motor vehicle accident, who presented to our hospital with diabetic ketoacidosis and clinical signs of pyelonephritis; subsequently, imaging demonstrated spontaneous bilateral renal subcapsular hematoma. Risk factors for the rare presentation in this patient included pyelonephritis, history of bilateral ureteral stent placement, and a remote history of a mild unilateral renal laceration secondary to a motor vehicle accident. Typically, patients with this condition achieve spontaneous resolution with conservative management. Our patient initially presented with diabetic ketoacidosis and pyelonephritis but gradually developed retroperitoneal bleeding and hemorrhagic shock. Our patient’s critical condition required close monitoring in an intensive care unit and a more invasive approach including unilateral left renal artery embolization followed by a unilateral left nephrectomy. The patient ultimately recovered and continued to be followed outpatient without any serious long-term complications.

## Introduction

Subcapsular renal hematoma is a rare condition that usually presents in the setting of acute trauma. Nontraumatic renal hematoma is rarer, and when they do occur, they usually have predisposing factors such as renal cyst rupture, renal tumor invasion, vascular abnormalities, and anticoagulated patients. Presentation is typically nonspontaneous with a clear etiology and is usually unilateral. We report a case of spontaneous bilateral subcapsular renal hematoma with unique imaging and discuss the possible etiology and our management approach.

## Case Presentation

**Figure 1.**
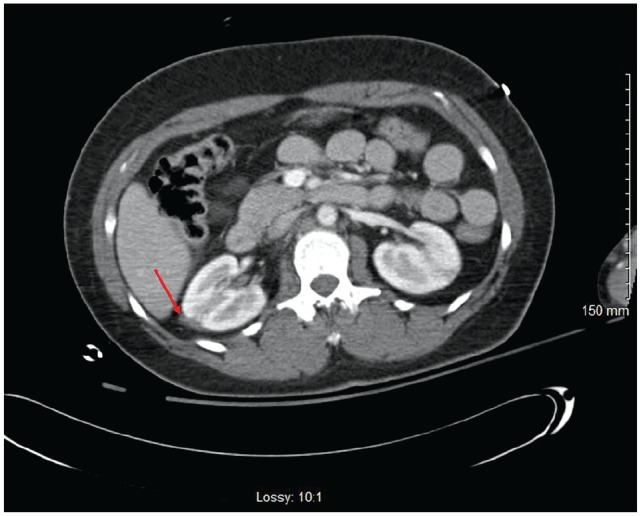
Computed tomography of abdomen with contrast demonstrating small amount of fluid in the right Gerota’s fascia (red arrow) with a subtle fracture of the upper pole of the right kidney.

**Figure 2.**
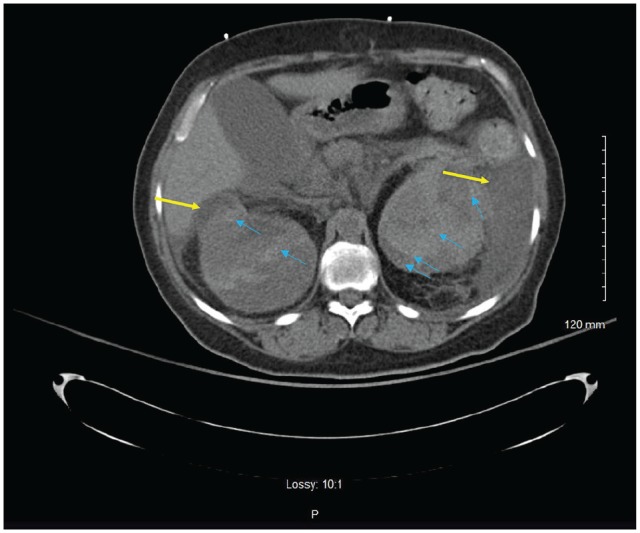
Computed tomography abdomen and pelvis without contrast showing evidence of large bilateral subcapsular renal hematomas (yellow arrows), left greater than right, with attenuation within these hematomas concerning for acute intermittent bleeds (blue arrows).

**Figure 3.**
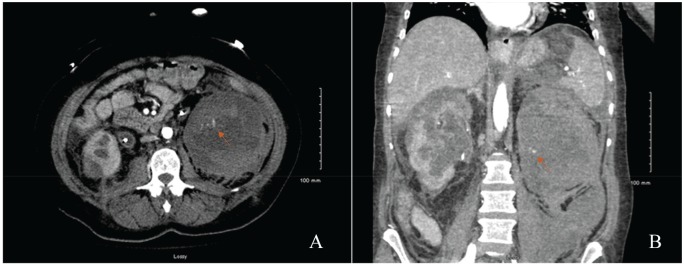
Triple-phase computed tomography of abdomen (A) and pelvis (B) demonstrates progression of the left-sided subcapsular hematoma measuring 10.7 × 10.7 ×16.8 cm with visualization of arterial extravasation on the left kidney (orange arrows).

A 51-year-old female presented to the emergency department for complaints of diffuse abdominal pain as well as left-sided flank pain for 1 week with associated nausea and vomiting. Patient denied any recent traumatic events. Past medical history is significant for uncontrolled diabetes mellitus type 2, bipolar disorder, and a history of motor vehicle accident 7 years prior that resulted in traumatic brain injury and residual mild cognitive impairment, pelvic fractures status-post open reduction with internal fixation, and a right-sided renal laceration that was managed conservatively (Figure 1). On presentation to the emergency department, urinalysis demonstrated hematuria with white blood cell clumps and frank pyuria in the setting of leukocytosis that was concerning for pyelonephritis. Furthermore, laboratory results demonstrated diabetic ketoacidosis (DKA). The patient was started on a DKA protocol with an insulin drip, as well as ceftriaxone 1 g daily for pyelonephritis. Within a few hours of admission, her left flank pain worsened, and the patient became tachycardic to 120 seconds, hypotensive with mean arterial pressure of 55 to 60 mm Hg, pale, and diaphoretic with worsening mental status. Hemoglobin decreased from 9.5 g/dL to 5.5 g/dL within 4 hours and lactate increased from 2.6 to 3.8 despite 4 L of fluid resuscitation. Antibiotics were broadened from ceftriaxone to linezolid and piperacillin-tazobactam, given her acute kidney injury. Resuscitation included transfusion of 1 unit packed RBC, fluids, and the initiation of norepinephrine for vasopressor support. Computed tomography (CT) of abdomen and pelvis without contrast demonstrated evidence of large bilateral subcapsular renal hematomas, left greater than right, with attenuation within these hematomas concerning for acute intermittent bleeds (Figure 2). The CT also showed a left-sided retroperitoneal hematoma measuring 10.3 × 4.6 × 12.1 cm with acute hemorrhage, as well as 2 ureteral stents of unknown age or origin. Repeat physical examination did not show any signs of trauma. One hour later, CT angiography of the abdomen and pelvis with triple-phase contrast to localize the source of bleeding showed that the left-sided subcapsular hematoma appears to have increased in size from 8.4 × 7.8 × 15 cm on the prior CT to 10.7 × 10.7 ×16.8 cm, as well as evidence of active arterial extravasation from the left renal artery into the retroperitoneum (Figure 3). The patient was then intubated and underwent an emergent embolization of the left renal artery by interventional radiology, which aided in hemodynamic stability along with placement of a perinephric drain. Patient was then started on hemodialysis as needed for uremia. On hospital day 2, blood cultures resulted positive for extended spectrum β-lactamase *Escherichia coli*; consequently, piperacillin-tazobactam was discontinued and meropenem 1 g intravenously every 12 hours was initiated. She underwent replacement of bilateral ureteral stents considering their unknown origin and the possibility of the stents being the source of the infection. On hospital day 3, she underwent IR-guided left-sided percutaneous drainage of necrotic tissue that developed after left renal artery embolization. A left nephrostomy was placed for continuous drainage. Intraoperative cultures from the perinephric drain that was previously placed during the embolization procedure were positive for *Candida albicans* and fluconazole was started. Patient continued to experience febrile episodes and on hospital day 6, the left-sided nephrostomy tube was upsized, and an additional drain was placed in the left iliopsoas to drain the retroperitoneal hematoma. Furthermore, an additional nephrostomy and drain was placed in the right kidney for hydronephrosis and hematoma evacuation. Fluid from all of the drains produced serosanguineous fluid. The patient’s clinical course continued to improve and was then transferred to the medical floor. The drains were eventually removed when the total output was <60 mL per 24-hour period. Patient received hemodialysis as needed for symptomatic uremia for the following 6 weeks. Eventually, the patient’s condition stabilized enough so that she no longer needed hemodialysis and underwent a left radical nephrectomy (adrenal-sparing) and left partial ureterectomy. Anatomic pathology of the removed kidney did not show any evidence for malignancy or any other significant findings. She subsequently remained stable and hemodialysis independent.

## Discussion

Spontaneous bilateral subcapsular hematomas are uncommon, though life threatening. In 1856, Wunderlich described Lenk’s triad including acute flank pain, tenderness, and symptoms of internal bleeding, which were common symptoms in patients with spontaneous subcapsular renal hematoma.^1^ The most frequently identified etiologies include renal neoplasm 61.5% cases, in which angiomyolipoma and renal cell carcinomas are leading causes, followed by vasculitis in 17%, 2.4% due to infection, and 6.7% were idiopathic.^2^

The literature on the topic is sparse, limited to case reports. A case report by Mckinnon et al describes a patient with both polyarteritis nodosa and microscopic polyangiitis (overlap syndrome) that developed spontaneous bilateral subcapsular hematoma.^3^ The patient was a poor surgical candidate due to numerous aneurysms within the kidneys, so she was treated medically with cyclophosphamide and steroids. She responded well to immunosuppressants and was discharged home 3 weeks after admission.^3^ Lal et al report a patient who has metastatic choriocarcinoma to bilateral kidneys who developed this rare condition and was managed successfully with angioembolization with polyvinyl alcohol particles. The patient’s condition was further complicated with simultaneous jejunal metastasis, nosocomial pneumonia that led to sepsis and death.^4^ Antiplatelet drug therapy might also play a role as a contributing factor possibly more so during pregnancy.^5^ Other factors include anticoagulation, nonsteroidal anti-inflammatory drugs, cystic diseases, blood dyscrasia, hypertension, hemodialysis, post-lithotripsy, or ureterorenoscopy.^6,7^

In our case, the patient presented with DKA and hemorrhagic shock. Due to her hemodynamic instability, as well as severe metabolic derangements, she was not a candidate for surgical intervention; however, after consultation with interventional radiology, the patient underwent a catheter-directed coil angioembolization of the left renal artery to terminate active arterial extravasation. Once the patient was stabilized and no longer requiring hemodynamic support, the nephrectomy was performed.

The patient had multiple factors that were believed to set the stage for bilateral subcapsular hematoma. To begin, uncontrolled diabetes mellitus predisposes her to diabetic nephropathy and a urinary tract infection, especially pyelonephritis. Her condition was further complicated by having active bleed and hemorrhagic shock. Furthermore, the patient was found to have bilateral ureteral stents in place for an unknown reason and for an unknown duration of time. Due to her decreased cognitive function and history of traumatic brain injury, she was unable to provide information for the indication of the previous ureteral stents. In addition, the patient had a history of a minor laceration of the right kidney as a result of a motor vehicle accident 7 years prior, which could be a potential contributing factor to her clinical presentation. Despite all of these risk factors, which could have led to the presentation, none explain the acute and bilateral nature.

Imaging modality of choice for the condition usually involves ultrasound for rapid identification of the condition. However, ultrasound sensitivity and specificity is operator dependent and confirmation CT should follow abnormal ultrasound results.^2^ CT scan has high sensitivity and specificity and can help identify the underlying cause.^2^ Magnetic resonance imaging is an alternative to CT, and it can differentiate blood collection from masses as well as identify small tumors.^8^ Angiography may be useful for the diagnosis of vascular diseases and when embolization is required. On literature review, in cases where etiology cannot be determined, patients with stable conditions or benign disease, the management is focused on nonsurgical approach that includes antibiotics, pain control, monitoring vital signs, and frequent hemoglobin levels. Follow-up CT scan should be performed at 3-month intervals until hematoma resolves. In cases where patients are hemodynamically unstable, or when the underlying etiology is renal cell carcinoma, exploratory surgery or nephrectomy can take place that is what took place in our case.

## Conclusion

Spontaneous subcapsular hematoma is a rare entity with rare imaging as described and illustrated in our patient. The main interest of this case study lies in bilateral spontaneous subcapsular hematoma in a patient who has multiple factors that could play a role in the presenting condition. The primary etiology cannot be determined; however, our management included transcatheter coil angioembolization, bilateral nephrostomies, and eventually a nephrectomy of the left kidney. We present the first case report of spontaneous bilateral subcapsular hematoma and despite multiple risk factors, none explained the acute and unique presentation of our patient.
